# Proteomic changes to immune and inflammatory processes underlie lung preservation using *ex vivo* cytokine adsorption

**DOI:** 10.3389/fcvm.2023.1274444

**Published:** 2023-10-02

**Authors:** Anna Niroomand, Gabriel Hirdman, Leif Pierre, Haider Ghaidan, Sven Kjellström, Martin Stenlo, Snejana Hyllén, Franziska Olm, Sandra Lindstedt

**Affiliations:** ^1^ Department of Clinical Sciences, Lund University, Lund, Sweden; ^2^ Lund Stem Cell Center, Lund University, Lund, Sweden; ^3^ School of Medicine, Rutgers Robert Wood Johnson University, New Brunswick, NJ, United States; ^4^ Wallenberg Center for Molecular Medicine, Lund University, Lund, Sweden; ^5^ Department of Cardiothoracic Surgery and Transplantation, Skåne University Hospital, Lund, Sweden; ^6^ Department of Cardiothoracic Anesthesia and Intensive Care, Skåne University Hospital, Lund, Sweden; ^7^ Department of Clinical Sciences, BioMS, Lund University, Lund, Sweden

**Keywords:** lung transplantation, mass spectrometry, *ex vivo* lung perfusion, cytokine adsorption, proteomics

## Abstract

**Introduction:**

In recent years, the field of graft preservation has made considerable strides in improving outcomes related to solid organ restoration and regeneration. Ex vivo lung perfusion (EVLP) in line with the related devices and treatments has yielded promising results within preclinical and clinical studies, with the potential to improve graft quality. Its main benefit is to render marginal and declined donor lungs suitable for transplantation, ultimately increasing the donor pool available for transplantation. In addition, using such therapies in machine perfusion could also increase preservation time, facilitating logistical planning. Cytokine adsorption has been demonstrated as a potentially safe and effective therapy when applied to the EVLP circuit and post-transplantation. However, the mechanism by which this therapy improves the donor lung on a molecular basis is not yet fully understood.

**Methods:**

We hypothesized that there were characteristic inflammatory and immunomodulatory differences between the lungs treated with and without cytokine adsorption, reflecting proteomic changes in the gene ontology pathways and across inflammation-related proteins. In this study, we investigate the molecular mechanisms and signaling pathways of how cytokine adsorption impacts lung function when used during EVLP and post-transplantation as hemoperfusion in a porcine model. Lung tissues during EVLP and post-lung transplantation were analyzed for their proteomic profiles using mass spectrometry.

**Results:**

We found through gene set enrichment analysis that the inflammatory and immune processes and coagulation pathways were significantly affected by the cytokine treatment after EVLP and transplantation.

**Conclusion:**

In conclusion, we showed that the molecular mechanisms are using a proteomic approach behind the previously reported effects of cytokine adsorption when compared to the non-treated transplant recipients undergoing EVLP.

## Introduction

The improvement of graft preservation remains a key goal in lung transplantation, with the ultimate objective of increasing the number and quality of transplants. Graft preservation is a key topic given the necessity of maintaining the quality of the harvested organ after it has been removed from the donor and remains in a vulnerable state, subject to ischemic damage. *Ex vivo* lung perfusion (EVLP) is a platform for evaluating and potentially treating donor lungs using machine perfusion with ventilation and perfusion support. This system was initially used for evaluating lung function in lungs from uncontrolled donation after circulatory death and later for evaluating marginal donor lungs, with the Lund group performing the first transplant using marginal lungs after EVLP in 2005 (1–5). Subsequent development has allowed for a stable EVLP protocol that can run for hours and evaluate suboptimal lungs (6, 7). The system also allows for implementing targeted therapies that could be used to recondition or ameliorate marginal and damaged lungs (8). The benefits of delivering a specific intervention during EVLP include using an isolated system in which the treatment can be administered directly to the target organ, bypassing any effects treatment may have in the systemic circulation.

The restoration or regeneration of damaged organs is a particularly important goal given the number of lungs that are declined for transplantation due to poor quality. Donor organ availability limits the number of possible transplantations, resulting in waiting list mortality. An estimated 60% of donor lungs are rejected after evaluation for acceptance, with the fear that damaged lungs will result in higher complication rates (9, 10). Refusal of lung grafts can caused by acute lung injury (ALI) stemming from several etiologies, such as aspiration, infection, trauma, and neurogenic edema, with the most severe form of ALI manifesting as acute respiratory distress syndrome (ARDS) (11).

Thus, EVLP allows for treating lungs that are damaged by ALI and ARDS to recuperate them for transplantation. Several potential therapies have been tested for graft preservation using EVLP, such as the use of mesenchymal stem cells, dialysis, and cytokine adsorption, to name a few. In particular, cytokine adsorption has been investigated given the established significance of cytokines in mediating inflammatory processes. Adsorbers rely on polymer beads that target the middle and low-molecular-weight molecules, which have been shown to reduce cytokine levels in severe sepsis (12–16). They have also been used in other transplantation types, such as orthotopic heart and kidney transplants (17, 18). We previously reported on the use of cytokine adsorption in a porcine model of transplantation (19) and human transplants (20). In recipients with lungs treated during EVLP and post-transplantation, inflammation and pulmonary function improved, and the incidence of primary graft dysfunction was reduced. The mechanisms and pathways involved in graft improvement with cytokine adsorption both from this and other EVLP studies have not been fully elucidated. Data on the proteomic profiles characterizing lung transplantation using tissue are extremely limited, particularly from models after the transplant in the recipients and none as far as we identified specific to cytokine adsorption (21, 22). We hypothesized that a proteomic approach would demonstrate what pathways were altered by the treatment. Using lung biopsies obtained during EVLP and post-transplantation in our model, we compared the treated grafts to the non-treated grafts and compared both to the normal lungs, finding significant alterations to pathways related to immune and inflammatory processes.

## Methods

### Porcine model

#### Ethical considerations for porcine experiments

The study was approved by the local Ethics Committee for Animal Research (DNR 5.2.18-4903/16 and DNR 5.2.18-8927/16) of the Lund University. All animals received care according to the USA Principles of Laboratory Animal Care of the National Society for Medical Research, Guide for the Care and Use of Laboratory Animals, National Academies Press (1996).

### Animal preparation

A total of 24 male and female adult farm-raised wild-type American Yorkshire pigs (*Sus scrofa domesticus*) were used in this study, with 12 designated as donors and 12 designated as recipients. These animals were previously described in a prior publication, which includes alternate variables and outcomes that were not re-reported in this publication (19). Seraclone™ Anti-A blood grouping reagent (Bio-Rad, Medical Diagnostics GmbH, Dreieich, Germany) was used to determine the blood types of animals prior to the experiment, and these animals were then matched as donor–recipient pairs according to blood type and weight. Premedication, preparation, and ventilatory settings were previously described (19), such as xylazine (Rompun vet. 20 mg/kg, Bayer AG, Leverkusen, Germany), ketamine (Ketaminol vet. 20 mg/kg, Farmaceutici Gellini S.p.A., Aprilia, Italy), peripheral line insertion, urinary catheter, and endotracheal intubation. Mechanical ventilation (Servo 900C, Siemens, Solna, Sweden) was set to volume-controlled ventilation with a 1:2 inspiration-to-expiration ratio and a tidal volume of 6–8 ml/kg. All animals were monitored with a pulmonary artery catheter (Swan-Ganz CCOmbo V and Introflex, Edwards Lifesciences Services GmbH, Unterschleissheim, Germany).

### ARDS induction in donor animals

ARDS was induced using lipopolysaccharide (LPS) from the Gram-negative bacterium *Escherichia coli* (O111:B4, Sigma Aldrich, Merck KGaA, Darmstadt, Germany), as previously described (19). The endotoxin was administered as an infusion diluted in saline over 1 h (2 µg/kg/min) and then at a lower rate for another 1 h. All animals developed hemodynamic instability, which was treated with a continuous infusion of norepinephrine (40 µg/ml, 0.05–2 µg/kg/min) (Pfizer AB, Sollentuna, Sweden) and dobutamine (2 mg/ml, 2.5–5 µg/kg/min) (Hameln Pharma Plus GmbH, Hameln, Germany). Fluid loss was compensated with Ringer's acetate (Baxter Medical AB, Kista, Sweden). A total volume of 6 ml of blood sample was collected before induction of lung injury and thereafter every hour from the injured donor who was used for the analyses as previously reported (19). In addition, 1 ml of blood sample was collected for blood gas measurements every 30 min.

The Berlin criteria were used to define the ARDS stage based on the PaO_2_/FiO_2_ ratio taken using arterial blood gases. Blood gases were analyzed every 30 min (ABL 90 FLEX blood gas analyzer, Radiometer Medical ApS, Brønshøj, Denmark) and were normalized to a blood temperature of 37°C. Pulmonary harvest only proceeded after two arterial blood gases taken 15 min apart demonstrated a PaO_2_/FiO_2_ ratio of less than 300 mmHg and an absence of heart failure.

### Pulmonary harvest, EVLP, and left lung transplantation in the recipients

Pulmonary harvest, EVLP, and transplantation have been previously described in detail (19). In brief, pulmonary harvest was performed according to clinical practice and proceeded in an *en bloc* fashion through a median sternotomy and cannulation of the pulmonary artery and clamping of the superior vena cava, inferior vena cava, and ascending aorta. The lungs were anterogradely flushed with cold Perfadex® PLUS solution (XVIVO perfusion, Gothenburg, Sweden).

The *en bloc* lungs were placed on a Vivoline LS1 (XVIVO perfusion, Gothenburg, Sweden) for EVLP with a target perfusion of 40% of cardiac output and a tidal volume of 7 ml/kg of the donor body weight (23, 24). The system was primed with Steen™ Solution (XVIVO perfusion) and set to a circuit hematocrit of 15%–20% using donor red blood cells collected prior to ARDS induction. An additional Steen solution was added when the perfusate levels in the reservoir dropped below 300 ml. The lungs were cooled to 8–12°C for approximately 45 min before transplantation.

The left lung transplantation was performed according to clinical practice and as previously described, including the post-transplantation follow-up and immunosuppression (19). All animals were immunosuppressed using tacrolimus (0.15 mg/kg, PO) (Sandoz AS, Copenhagen, Denmark) and methylprednisolone (1 mg/kg, intravenously, twice daily) (Solumedrol, Pfizer, New York, USA)*.* Bronchoscopy was used to confirm an open bronchial anastomosis.

### Recipient follow-up and right pneumonectomy on the third day post-transplantation

The animals were kept under anesthesia for 3 days as previously described in detail (19). The recipient ventilatory strategy included the use of the lowest possible pressures while maintaining adequate oxygenation and ventilation with a positive end-expiratory pressure between 5 and 10 cmH_2_O and a peak pressure below 30 cmH_2_O. Arterial blood gases were monitored every hour post-transplantation. The pulmonary hilum was dissected through a mid-sternotomy, and a pneumonectomy of the right lung and accessory lobe allowed for an isolated assessment of the transplanted left lung. The recipient was followed for an additional 4 h during single-lung ventilation, and the tidal volume and respiratory rate were adjusted to maintain a peak pressure <30 cmH_2_O.

### Treatment with cytokine adsorption

As previously described (19), an adsorbent filter (CytoSorb®, CytoSorbents Europe GmbH, Berlin, Germany) was used to continuously filter the perfusate during EVLP through a veno-venous shunt from the reservoir at a rate of 300 ml/min. The filter was in place throughout EVLP and was followed up post-transplantation with an additional 12 h of extracorporeal hemoadsorption connected to the cytokine adsorber via a veno-venous shunt using a hemodialysis catheter (Power-Trialysis® Slim-Cath™, Becton, Dickinson and Company, NJ, USA) inserted in the jugular vein. The roller pump ran at a rate of 300 ml/min (19).

### Mass spectrometry analysis

#### Sample preparation and protein digestion

Biopsies were taken from the right lung after intubation as baseline samples with random selection from both the treated and non-treated groups (*n* = 6 baseline samples). Biopsies were also obtained from the right lung after 4 h of EVLP and from the left lung post-transplantation on day 3 of observation from both the treated and non-treated groups (*n* = 6 per baseline, treated EVLP, non-treated EVLP, and non-treated post-transplant groups and *n* = 5 for treated post-transplant group after analysis). Proteins were extracted from homogenized tissues and were solubilized in a 2% sodium dodecyl sulfate (SDS, Sigma Aldrich, Darmstadt, Germany). A bicinchoninic acid (BCA) assay (Pierce, Thermo Fisher Scientific, Waltham, MA, USA) was performed to determine protein concentration. Subsequently, 30 µg of protein was digested using an S-Trap digestion protocol. Samples were reduced with 20 mM dithiothreitol (DTT, Sigma Aldrich) for 45 min at 56°C and then incubated with 40 mM iodoacetamide (IAA, Sigma Aldrich) in the dark at room temperature for 30 min. Samples were acidified with 2.5% phosphoric acid and washed with buffer before binding to an S-Trap CO_2_-micro-80 column (ProTifi, Fairport, New York, USA) and were double digested overnight at 37°C with lysine-C (Promega mass spec grade at a 1:50 ratio of enzyme to protein by ng, Promega, Madison, WI, USA) and trypsin (Promega sequence grade at a 1:50 ratio of enzyme to protein by ng).

### Peptide mixing and pre-fractionation

Fractionation was carried out using a Waters XBridge BH130 C18 3.5 μm, 2.1 mm × 150 mm column, on an Ultimate 3000 rapid separation high-performance liquid chromatography (RS HPLC) (Thermo Scientific, Waltham, MA, USA) operating at 200 µl/min. The mobile phases were solvent A, 10 mM ammonium formate at pH 10, and solvent B, 90% acetonitrile and 10% water containing 10 mM ammonium formate at pH 10. Peptides were separated using the following gradients: 0 min 0% B; 3 min 0% B, 97 min 35% B; 98 min 80% B; and 108 min 80% B. The column was operated at RT, and the detection wavelength was 214 nm. We collected 96 fractions at 1 min intervals that were further concatenated to 48 fractions by combining two fractions that are 24 fractions apart, i.e., #1 and #25 and #2 and #26. The fractions were dried in the SpeedVac.

### LC-MS/MS data acquisition

#### DDA data acquisition on timsTOF Pro 2

The fractions were resuspended in 0.1% formic acid, and peptide determination was performed in a NanoDrop system (DeNovix, Wilmington, DE, USA) before liquid chromatography tandem mass spectrometry (LC-MS/MS) analysis. A total of 400 ng of each fraction was loaded on Evosep tips (Evosep Biosystems, Odense, Denmark) for separation with nanoflow reversed-phase chromatography with an Evosep One LC system (Evosep Biosystems). Separation was performed with the 30 SPD method (gradient length of 44 min) using a 15 cm × 150 µm Evosep column (Evosep Biosystems) packed with 1.5 μm ReproSil-Pur C18-AQ particles. The Evosep One was coupled to a timsTOF Pro 2 ion mobility mass spectrometer (Bruker, Billerica, MA, USA) operated in data dependent acquisition - parallel accumulation - serial fragmentation (DDA PASEF) with 10 PASEF scans per acquisition cycle and accumulation and ramp times of 100 ms each. Singly charged precursors were excluded, the “target value” was set to 20,000, and dynamic exclusion was activated and set to 0.4 min. The quadrupole isolation width was set to 2 Th for m/z < 700 and 3 Th for m/z > 800. All subsequent DDA files were used to build a spectral library in Fragpipe v 180 (25–28), with the following parameters, i.e., missed cleavages = 2, min peptide length = 7, max peptide length = 50, and common internal retention time peptides (CiRTs), that were used for spectral library retention time calibration. UniProt UP000008227 FASTA (release 2023_01) was used as a database with reversed target sequences as decoys. The generated library consisted of 10,296 protein groups in total. A py_diAID method (29) was generated by subjecting the 48 DDA fraction runs and was used to run all individual samples in the study in diaPASEF on the timsTOF Pro 2.

### DIA data acquisition on timsTOF Pro 2

The samples were loaded onto Evosep tips (Evosep Biosystems) and separated with the same gradient as for DDA data acquisition. MS data were acquired using the diaPASEF method. The accumulation and ramp times were set to 100 ms. DIA scans were acquired with 25 m/z isolation windows spanning 247–1,350 m/z and 0.60–1.60 1/K0 ion mobility ranges and an estimated cycle time of 2.76 s. The collision energy was ramped linearly as a function of the mobility from 59 eV at 1/K0 = 1.6 Vs cm^−2^ to 20 eV at 1/K0 = 0.6 Vs cm^−2^.

### Bioinformatic analysis of LC-MS/MS data

Raw LC-MS/MS data were analyzed using DIA-NN v 1.8.1. The quantification mode was set to Robust LC (high precision), with default RT-dependent normalization and the Fragpipe spectral library. Subsequently, the output files were loaded into RStudio v 2022.12.0 with R v 4.2.2. The MS-DAP package v 1.05 (30) was used for normalization and differential expression analysis. The identified proteins were first filtered at 75% of identifications per contrast. Normalization was performed using variance stabilizing normalization followed by mode between protein normalization. Differential expression analysis was performed using the DEqMS R package (31). Log(2)-fold change thresholds were inferred through bootstrapping in the MS-DAP package. Significantly changed proteins were defined as false discovery rate (FDR)-corrected *p*-values (*q*-values) of >0.05 and Log (2)-fold change of ±0.313, 0.344, and 0.354 for the non-treated vs. treatment, baseline vs. treatment, and baseline vs. non-treated groups, respectively. For the heatmap, the MaxLFQ values were normalized using *z*-scores and plotted using the pheatmap package v 1.0.12 with Euclidean clustering. Gene set enrichment analysis (GSEA) analysis was performed by examining all proteins found through bioinformatic analysis after the filtering and normalization steps using the clusterProfiler package v 4.4.4. The mass spectrometry proteomics data have been deposited to the ProteomeXchange Consortium via the PRIDE partner repository with the dataset identifier PXD044413.

### Calculations and statistics

Continuous variables were reported as median and interquartile range (IQR). Statistically significant differences between groups were tested with the Student's *t*-test when comparing the two groups and within groups using ANOVA when data were normally distributed. Most analyses were conducted with the Mann–Whitney test and the Kruskal–Wallis tests when data were not normally distributed. A chi-squared test was performed to analyze the observed frequencies of the categorical variables. These statistical analyses were performed using GraphPad Prism 9.1. The statistics used within mass spectrometry analysis are reported in the “Bioinformatic analysis of LC-MS/MS data” section. Significance was defined as *p* < 0.001 (***), *p* < 0.01 (**), *p* < 0.05 (*), and *p* > 0.05 (not significant).

## Results

### Mass spectrometry analysis showed significant differences in protein expression after cytokine adsorption treatment in EVLP

Lung biopsies obtained prior to injury induction were collected as baseline samples and subsequently compared to the lung biopsies obtained after EVLP from both the treated and non-treated groups. After induction of lung injury, we found no significant differences in the PaO_2_/FiO_2_ ratios between the treated (208.2 ± 55.5 mmHg) and non-treated groups (225.3 ± 33.6 mmHg, *p* = 0l733). By the end of EVLP, the treated lungs had increased their ratio to 324 ± 70 mmHg, whereas the non-treated lungs did not pass the clinical acceptance with a PaO_2_/FiO_2_ ratio of 249 ± 143 in the 10 pigs assigned to the non-treated group.

**Figure 1:**
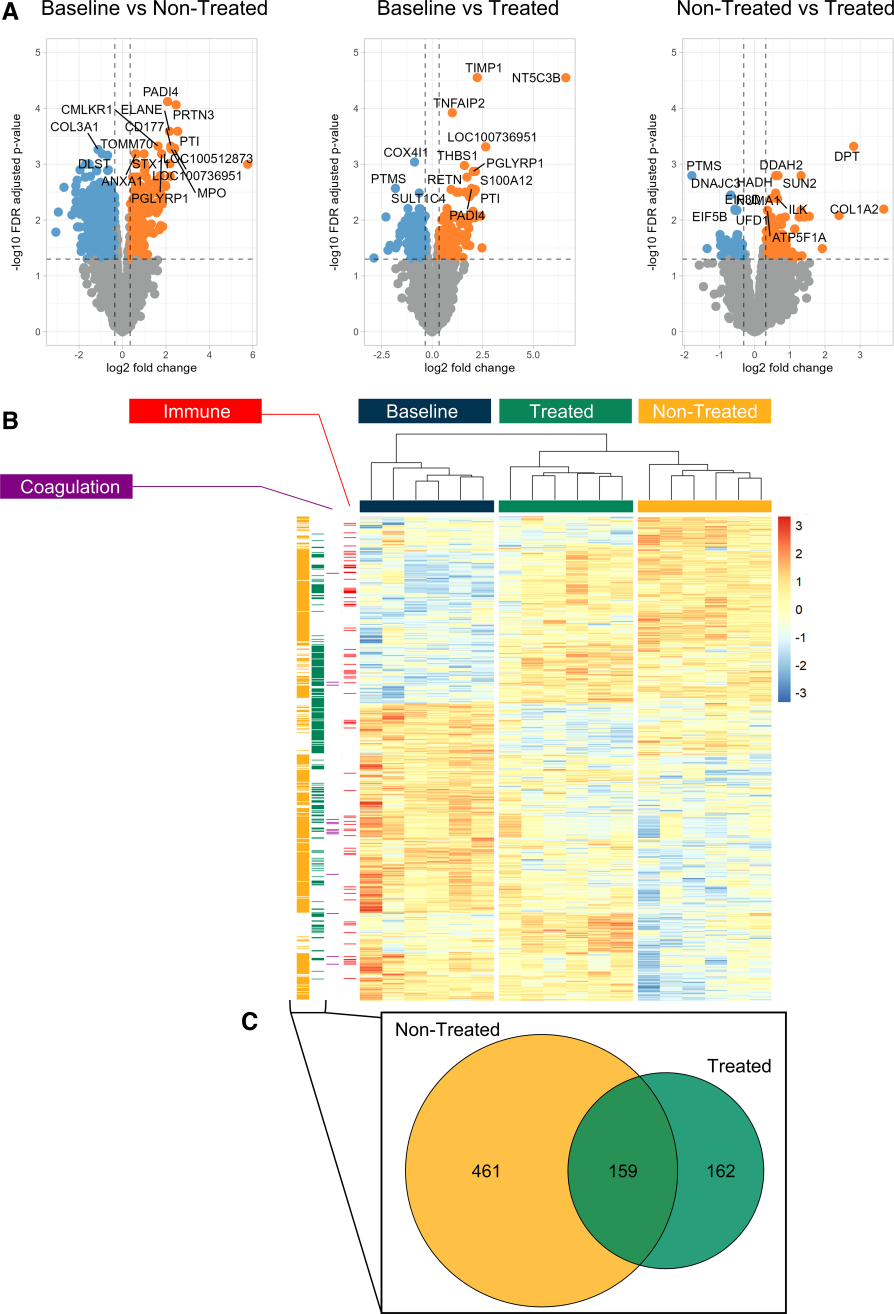
Treated and non-treated lungs could be clearly distinguished by their proteomic profiles based on differential expression of proteins found at the end of EVLP. Comparisons between the groups and those with normal lung tissue (baseline) showed significant differences in the overall distribution of protein expression. (**A**) Volcano plots of the proteins identified across the three comparison groups (non-treated vs. treated, baseline vs. treated, and baseline vs. non-treated). For those that were statistically significantly differentially expressed, blue marks the underexpressed proteins, and orange marks the overexpressed proteins within the comparisons. (**B**) Unsupervised hierarchical clustering was performed on the proteins to produce the heatmap across the three groups. GSEA showed pathways from the biological processes. The proteins from GO terms are highlighted in columns to the left of the heatmap: the immune system process pathway (red, GO Term GO:0002376) and coagulation (purple, GO:0007596). The proteins that were differentially expressed in the non-treated group compared to the baseline group are shown separately in the yellow column to the left of the heatmap. The differentially expressed proteins in the treated group are shown in the green column to the left. (**C**) Of those proteins that were differentially expressed between baseline vs. treated groups and baseline vs. non-treated groups, the Venn diagram demonstrates the distribution of unique or shared identities. All graphs represent data from the treated group (*n* = 6), non-treated group (*n* = 6), and baseline group (*n* = 6). Statistically significant differences are reported as FDR-corrected *p*-values (*q*-values) using log(2)-fold change differences between groups.

Figure 1A shows the volcano plots depicting the upregulated and downregulated proteins across the three comparison types. In the comparison between the baseline and the non-treated groups, 5,399 proteins were identified, of which 620 were statistically differentially expressed. Of those proteins that were differentially expressed, 373 were downregulated and 247 were upregulated. In the comparison between the baseline and the treated groups, 164 proteins were downregulated, and 157 were upregulated. Furthermore, in the comparison between the non-treated and treated groups, 57 proteins were downregulated and 112 were upregulated. Within the three groups, an unsupervised hierarchical clustering that was performed on the differentially expressed proteins demonstrated clustering of the groups (Figure 1B). The baseline, treated, and non-treated groups were discretely sorted based on the clustering of the proteins, showing the significantly different protein expression profiles at this point in EVLP.

In examining the clustering of protein expression profiles further, several comparison groups were considered separately and displayed to the left of the heatmap. This includes the significantly differentially expressed proteins found in the comparisons between the baseline and non-treated samples (yellow column, Figure 1B) and those between the baseline and treated samples (green column, Figure 1B). Of those proteins, the overlap in identities is displayed in the Venn diagram, as shown in Figure 1C. In addition, the specific pathways identified from the GSEA were highlighted to demonstrate the distribution of identified proteins across their locations in the heatmap (Figure 1B). Specifically, the immune system process pathway [gene ontology (GO) term GO:0002376] is displayed in the red column and blood coagulation (GO:0007596) in the purple column (Figure 1B). The immune system process term consists of a protein set size of 276 proteins and had a normalized enrichment score (NES) of 1.54 (*q*-value 0.028) when comparing the baseline and non-treated samples. Within the baseline and treated samples, the NES of the immune system process was 1.72 with a *q*-value of 0.027. For the term blood coagulation, when comparing the baseline group and the non-treated group, the pathway had an NES of −2.11 and a *q*-value of 0.002, and when comparing the baseline group and the treated group, the pathway had an NES of −1.84 and a *q*-value of 0.09.

**Figure 2:**
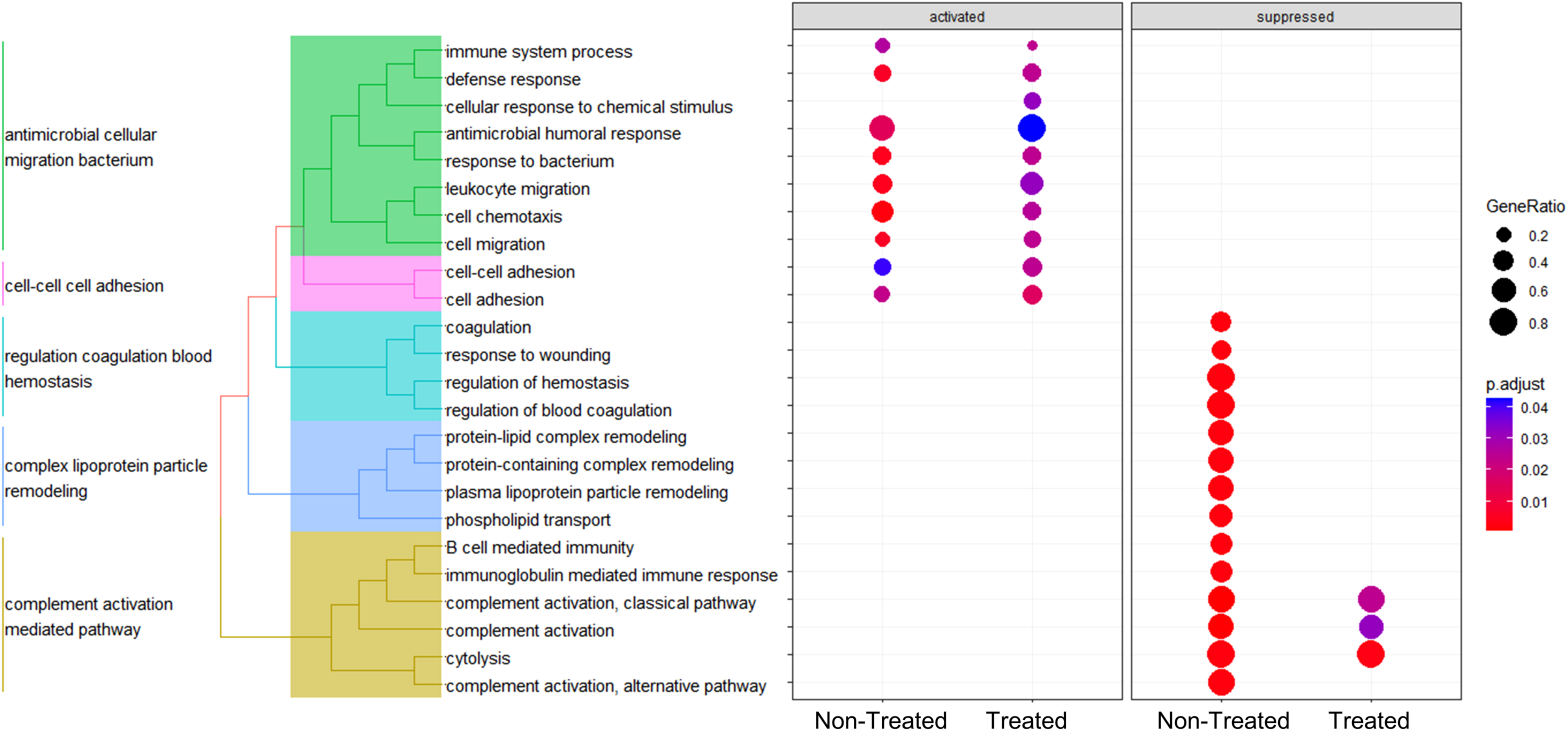
Key responses to injury were enriched in the treated and non-treated groups using GSEA during EVLP. Enriched pathways from the GSEA are shown for both the non-treated and treated groups in the dot plot. Unsupervised hierarchical clustering of the GO terms was performed to show the overarching biological processes involved. All graphs represent the data from the treated group (*n* = 6), non-treated group (*n* = 6), and baseline group (*n* = 6). Statistically significant differences are reported as FDR-corrected *p*-values (*q*-values) using log(2)-fold change differences between groups.

GSEA analysis across the comparisons between the baseline vs. non-treated and baseline vs. treated groups yielded enriched pathways, as shown in Figure 2. The hierarchical clustering was performed on the GO terms to demonstrate the overarching biological processes underscored by the GO terms. The clustering of enriched terms relied on pairwise similarities of the terms, and these terms were divided largely into five groups. These groups included mainly immune responses and responses to external stimuli, cell motility terms, coagulation terms, lipid transport, and leukocyte immunity, respectively.

**Figure 3:**
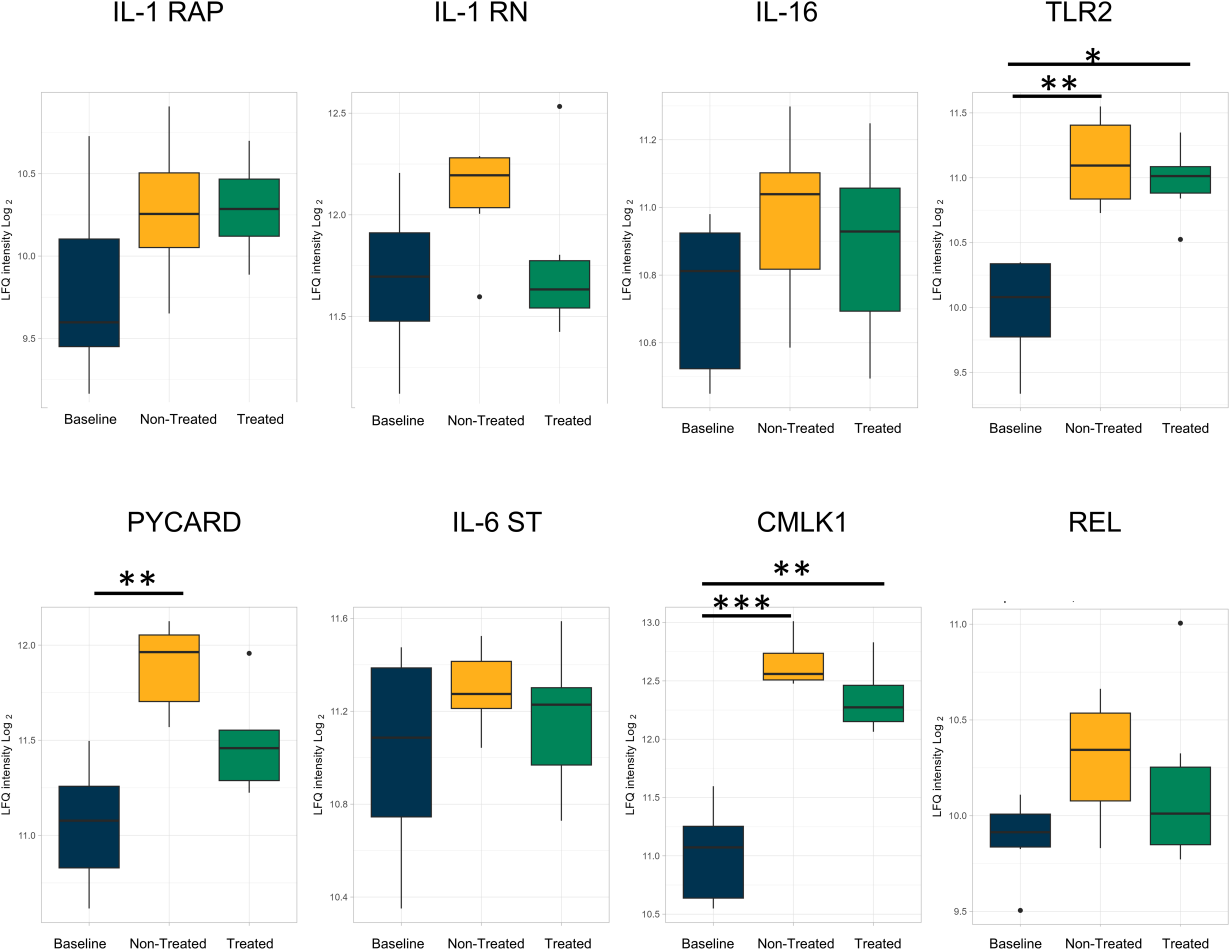
Proteins involved in inflammation and related to cytokine processes were upregulated in both the non-treated and treated groups compared to healthy lung (baseline) after EVLP; however, the extent of upregulation was attenuated by the treatment. All graphs represent data from the treated group (*n* = 6), non-treated group (*n* = 6), and baseline group (*n* = 6). The boxplots represent the median and interquartile range (box) with minimum and maximum values (whiskers). Statistically significant differences are reported as FDR-corrected *p*-values (*q*-values) using log(2)-fold change differences between groups. IL-1 RAP, interleukin-1 receptor accessory protein; IL-1 RN, interleukin-1 receptor antagonist protein; IL-16, pro-interleukin-16; TLR2, toll-like receptor 2; PYCARD, apoptosis-associated speck-like protein containing a CARD; IL-16 ST, interleukin-16 cytokine family signal transducer protein; CMLK1, chemerin-like receptor 1; REL, REL proto-onco, NK-kB subunit.

Individual proteins were identified for comparison across all three groups (Figure 3 and Table 1). Proteins related to inflammation and cytokine processes were found to be significantly increased in the non-treated group compared to those in the baseline group. These proteins include toll-like receptor 2 [TLR2, log(2)-fold change 1.14, *q*-value 0.007], apoptosis-associated speck-like protein containing a CARD [PYCARD, log(2)-fold change 0.84, *q*-value 0.005], and chemerin-like receptor 1 [CMLK1, log(2)-fold change 1.64, *q*-value 0.0005]. The interleukin-1 receptor accessory protein (IL-1 RAP) was numerically higher in the treated and non-treated groups, but this was not a statistically significant elevation [log(2)-fold change 0.50, *q*-value 0.25].

**Table 1:** Proteins identified related to the inflammation and cytokine processes compared between the treated and non-treated groups at the end of EVLP.

Protein	Log(2)-fold change	*q*-value
IL-1 RN	−0.27	0.41
IL-16	−0.22	0.24
TLR2	−0.06	0.86
PYCARD	−0.33	0.17
IL-6ST	−0.08	0.83
CMLK1	−0.22	0.42
REL	−0.19	0.57
AZU1	−0.08	0.92
PRTN3	−0.55	0.18
MPO	−0.003	0.99
S100A8	−0.21	0.69
DPP1	−0.28	0.07
ELANE	−0.15	0.76
CD163	−0.48	0.13
NOS	−0.20	0.66
NOSTRIN	0.45	0.07

IL-1 RN, interleukin-1 receptor antagonist protein; IL-16, pro-interleukin-16; TLR2, toll-like receptor 2; PYCARD, apoptosis-associated speck-like protein containing a CARD; IL-6ST, interleukin-6 cytokine family signal transducer; CMLK1, chemerin-like receptor 1; REL, REL proto-onco; AZU1, azurocidin; PRTN3, proteinase 3; MPO, myeloperoxidase; DPP1, dipeptidyl peptidase 1; ELANE, neutrophil-related elastase; NOS, nitric oxide synthase; NOSTRIN, nitric oxide synthase trafficking protein.

Several comparisons were not statistically significantly different but showed a numerical change between the treated and non-treated groups, such as the interleukin-1 receptor antagonist protein [IL-1 RN, log(2)-fold change −0.27, *q*-value 0.41], pro-interleukin-16 [IL-16, log(2)-fold change −0.22, *q*-value 0.24], and TLR 2 [log(2)-fold change −0.06, *q*-value 0.86]. Other observed numerical decreases in expression included PYCARD [log(2)-fold change −0.33, *q*-value 0.17], interleukin-6 cytokine family signal transducer [IL-6ST, log(2)-fold change −0.08, *q*-value 0.83], chemerin-like receptor 1 [CMLK1, log(2)-fold change −0.22, *q*-value 0.42], and REL proto-onco [REL, log(2)-fold change −0.19, *q*-value 0.57].

**Figure 4:**
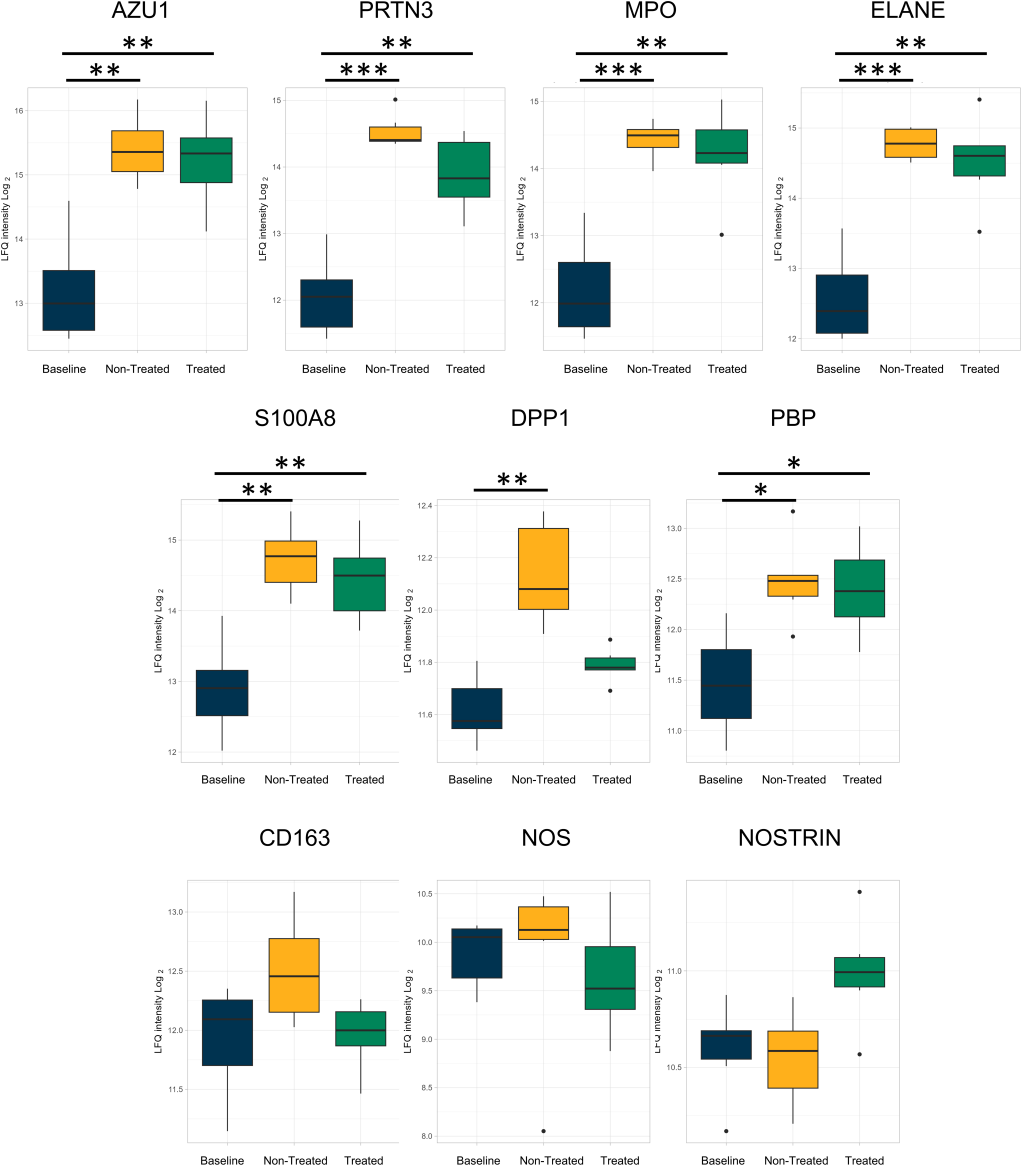
Neutrophil- and macrophage-related proteins were less upregulated in the treated group relative to the baseline group compared to the non-treated group as individually analyzed after EVLP. All graphs represent the data from the treated group (*n* = 6), non-treated group (*n* = 6), and baseline group (*n* = 6). The boxplots represent the median and interquartile range (box) with minimum and maximum values (whiskers). Statistically significant differences are reported as FDR-corrected *p*-values (*q*-values) using log(2)-fold change differences between. groups Azu1, azurocidin; PRTN3, proteinase 3; MPO, myeloperoxidase; ELANE, elastase, neutrophil expressed; DPP1, dipeptidyl peptidase 1; PBP, platelet basic protein; NOS, nitric oxide synthase; NOSTRIN, nitric oxide synthase trafficking.

In addition, numeric, though not statistically significant, decreases in proteins related to neutrophils (Figure 4) between the treated and non-treated groups were found. These proteins include azurocidin [AZU1, log(2)-fold change −0.08, *q*-value 0.92], proteinase 3 [PRTN3, log(2)-fold change −0.55, *q*-value 0.18], myeloperoxidase [MPO, log(2)-fold change −0.003, *q*-value 0.99], protein S100A8 [log(2)-fold change −0.21, *q*-value 0.69], dipeptidyl peptidase 1 [DPP1, log(2)-fold change −0.28, *q*-value 0.07], and neutrophil-related elastase [ELANE, log(2)-fold change −0.15, *q*-value 0.76]. Moreover, there were numeric decreases in proteins related to macrophage-related proteins, such as CD163 [log(2)-fold change −0.48, *q*-value 0.13] and nitric oxide synthase [NOS, log(2)-fold change −0.20, *q*-value 0.66]. This coincided with an increase in the nitric oxide synthase trafficking protein when comparing the treated and non-treated values [nitric oxide synthase trafficking inducer (NOSTRIN), log(2)-fold change 0.45, *q*-value 0.07].

### Pathways related to the immune system and inflammatory responses were significantly altered in the treated group after lung transplantation

Samples collected from the third day after the transplantation were compared between the non-treated and treated groups and additionally analyzed compared to the same baseline samples as previously described. By the end of the experiment, the PaO_2_/FiO_2_ ratio of the treated group (442.0 ± 90.2 mmHg) was significantly higher than that of the non-treated group (174.9 ± 31.0 mmHg, *p* = 0.0022).

**Figure 5:**
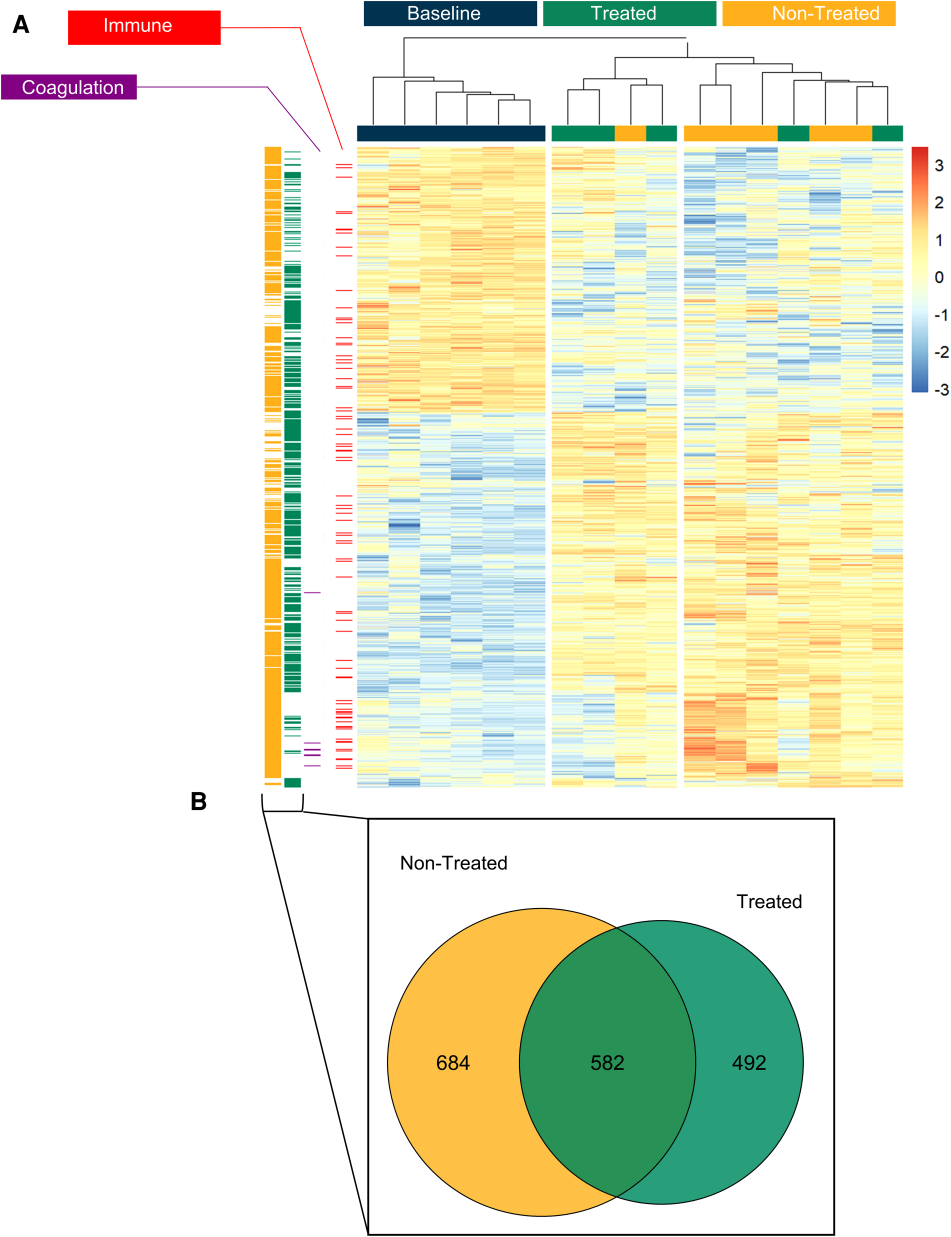
The treated group could be clearly differentiated from the non-treated group based on proteomic profiles the end of the post-transplant observational period and both demonstrated a difference from healthy lung tissue (baseline). (**A**) Unsupervised hierarchical clustering was performed on significantly differentially expressed proteins to produce the heatmap across the three groups (baseline, treated and non-treated). Gene set enrichment analysis (GSEA) showed pathways from the biological processes. The proteins from gene ontology (GO) terms are highlighted in columns to the left of the heatmap: the immune system process pathway (red, GO Term GO:0002376) and blood coagulation (purple, GO:0007596). The proteins which were differentially expressed in the non-treated group compared to the baseline are shown separately in the yellow column to the left of the heatmap. The differentially expressed proteins in the treated group are shown in the green column to the left. (**B**) Of those proteins which were differentially expressed between baseline vs treated and baseline vs non-treated, the Venn diagram demonstrates the distribution of unique or shared identities. All graphs represent data from the treated group (*n* = 5), non-treated group (*n* = 6), and baseline group (*n* = 6). Statistically significant differences are reported as FDR corrected p-values (*q*-values) using log(2)-fold change differences between groups.

Based on the expression profiles of the identified proteins, the hierarchical clustering again showed the separation of the groups into baseline, treated, and non-treated groups (Figure 5A). The same immune system process pathway and coagulation pathway were highlighted in separate columns to the left of the heatmap to demonstrate the location of the proteins identified within these sets. When comparing the treated and non-treated groups, the immune system process had an NES of −1.78 with a *q*-value of 5.22 × 10^−8^, whereas the stress response had an NES of 1.65 and a *q*-value of 5.21 × 10^−8^. Furthermore, the proteins differentially expressed in the baseline to non-treated comparison (yellow column, Figure 5A) and the baseline to treated comparison (green column, Figure 5A) were placed adjacent to the heatmap to show protein locations. From the expressed proteins, a greater extent of identity overlaps with 582 proteins in common can be found between the baseline to non-treated and baseline to treated comparisons, as shown in the Venn diagram in Figure 5B.

**Figure 6:**
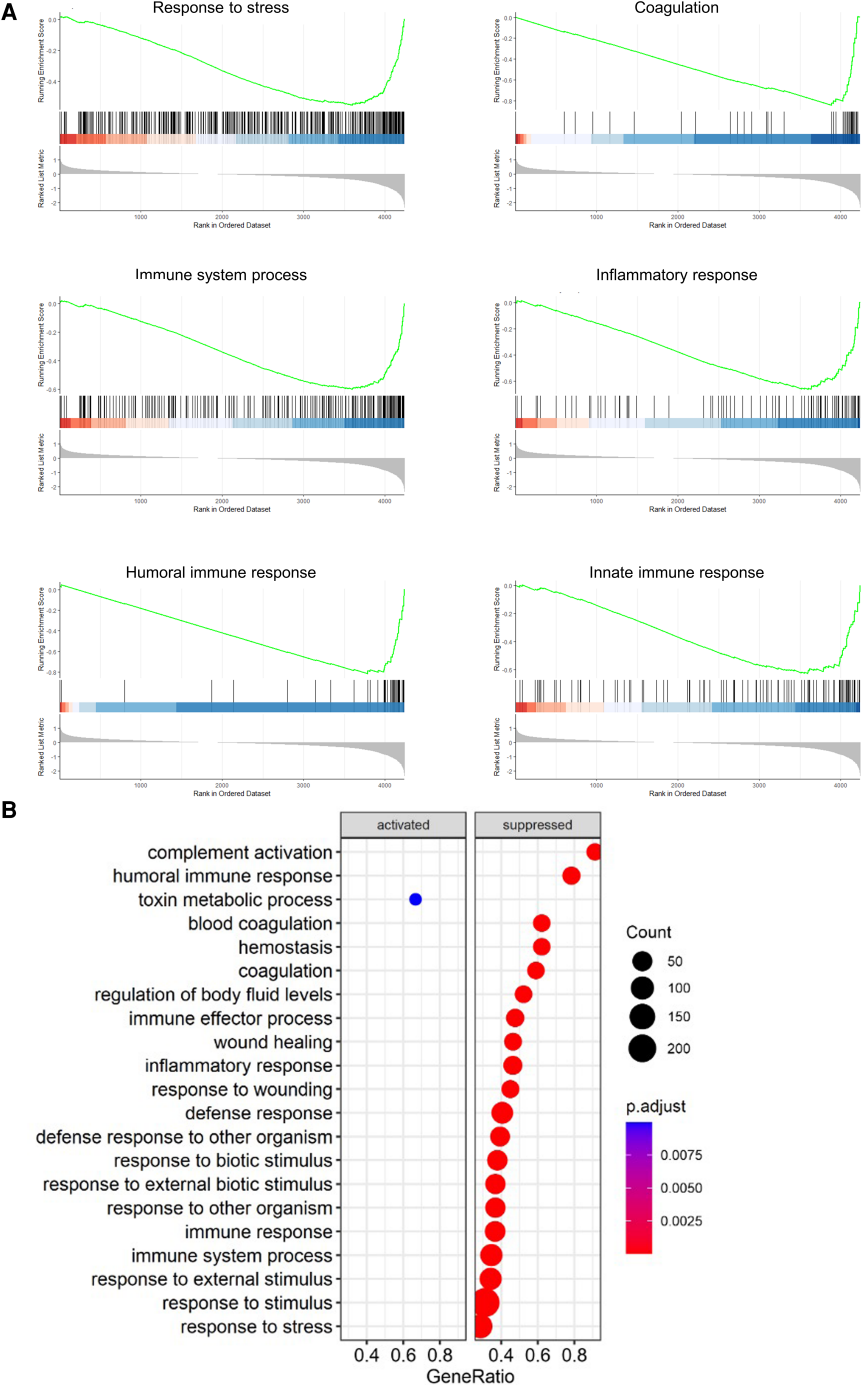
Pathways related to immune, inflammatory, and coagulative responses were suppressed in the treated group compared to the non-treated using GSEA after the end of post-transplantation observation. The pathways that were enriched between the non-treated vs. treated comparison are shown as a dot plot. All graphs represent the data from the treated group (*n* = 5), non-treated group (*n* = 6), and baseline group (*n* = 6). Statistically significant differences are reported as FDR-corrected *p*-values (*q*-values) using log(2)-fold change differences between groups.

GSEA analysis of the proteins found within the comparisons showed a number of similar pathways as found within the EVLP. Several pathways were identified as either activated or suppressed within the non-treated vs. treated comparison (Figure 6). The only statistically significantly activated process was the term “toxin metabolic process.” All others were suppressed in the treated group, which included pathways involving immune responses, stress responses, wound healing, and defense responses. In addition, blood coagulation and hemostasis were identified as suppressed pathways.

**Figure 7:**
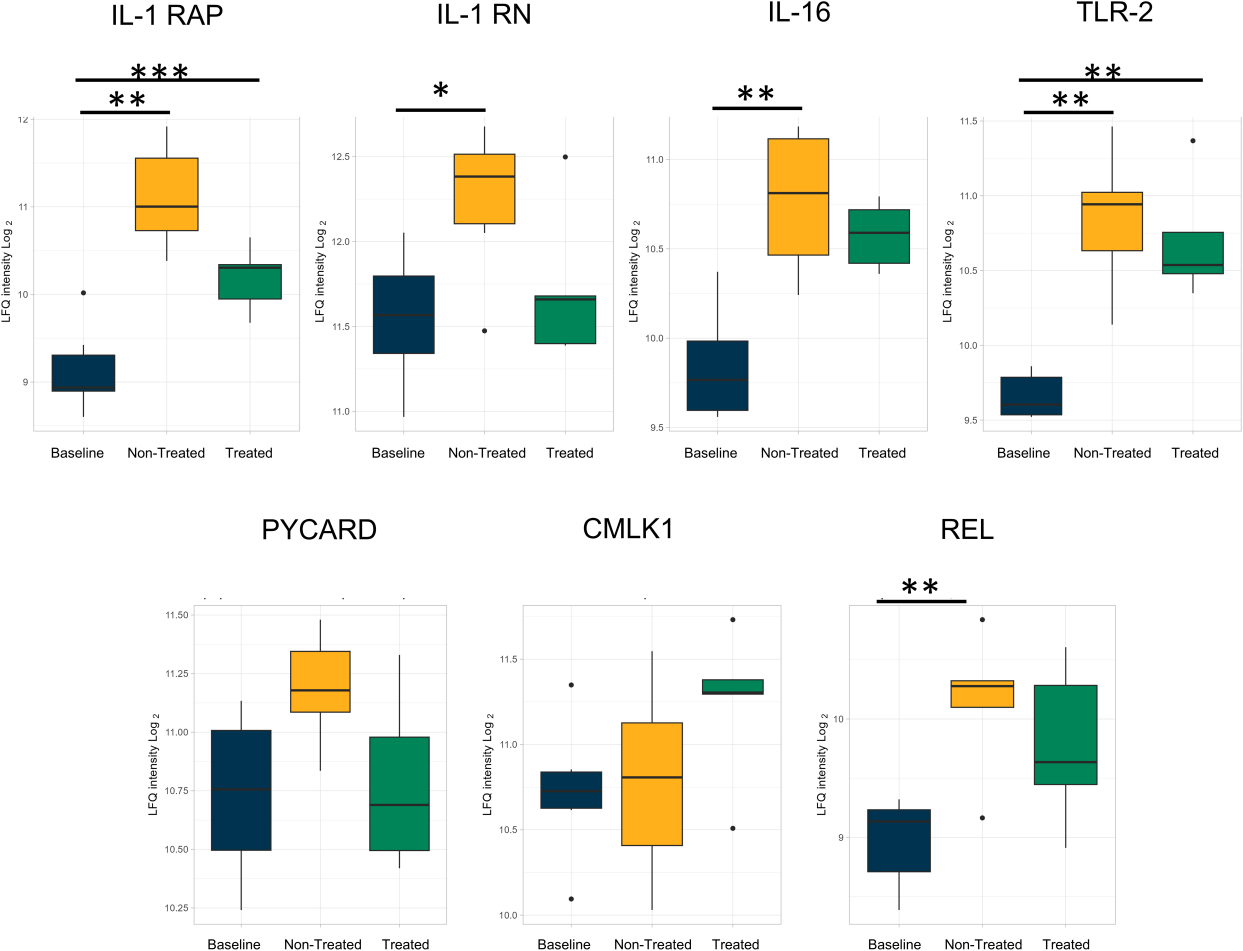
By the end of the post-transplantation observational period, the same reduction of proteins involved in inflammation and related to cytokine processes in the treated group was observed after cytokine adsorption in EVLP. All graphs represent the data from the treated group (*n* = 5), non-treated group (*n* = 6), and baseline group (*n* = 6). The boxplots represent the median and interquartile range (box) with minimum and maximum values (whiskers). Statistically significant differences are reported as FDR-corrected *p*-values (*q*-values) using log(2)-fold change differences between groups. IL-1 RAP, interleukin-1 receptor accessory protein; IL-1 RN, interleukin-1 receptor antagonist protein; IL-16, pro-interleukin-16; TLR2, toll-like receptor 2; PYCARD, apoptosis-associated speck-like protein containing a CARD; IL-16 ST, interleukin-16 cytokine family signal transducer protein; CMLK1, chemerin-like receptor 1; REL, REL proto-onco, NK-kB subunit.

**Figure 8:**
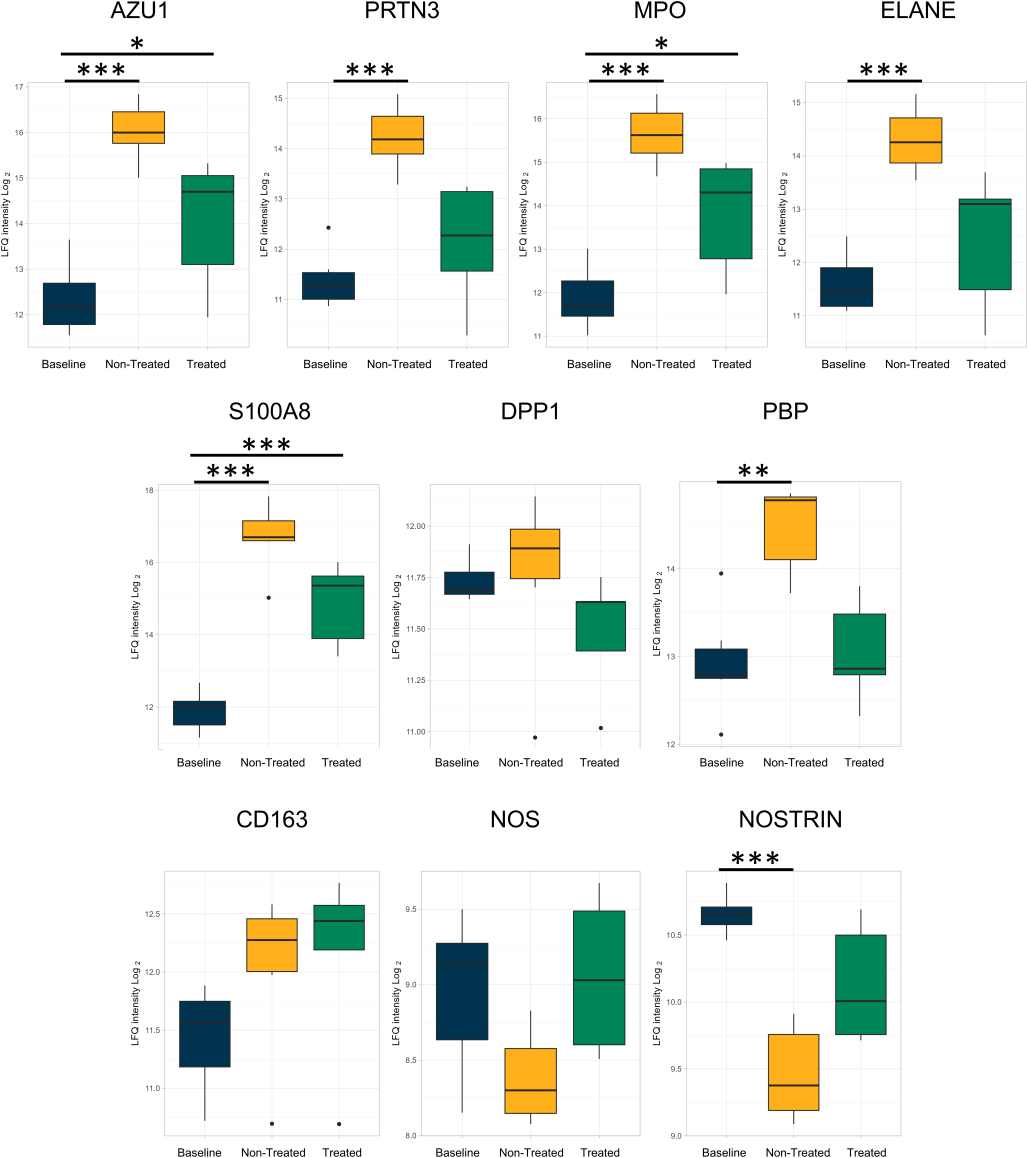
Proteins related to neutrophils and macrophage processes that were reduced after *ex vivo* lung perfusion were again reduced in the treated group when compared to the non-treated group after the end of the post-transplantation observation period. All graphs represent the data from the treated group (*n* = 5), non-treated group (*n* = 6), and baseline group (*n* = 6). The boxplots represent the median and interquartile range (box) with minimum and maximum values (whiskers). Statistically significant differences are reported as FDR-corrected *p*-values (*q*-values) using log(2)-fold change differences between groups. Azu1, azurocidin; PRTN3, proteinase 3; MPO, myeloperoxidase; ELANE, elastase, neutrophil expressed; DPP1, dipeptidyl peptidase 1; PBP, platelet basic protein; NOS, nitric oxide synthase; NOSTRIN, nitric oxide synthase trafficking.

The individual proteins compared after EVLP were again examined after transplantation (Figures 7, 8 and Table 2). Between the treated and non-treated groups, decreases in proteins involved in inflammation were observed, such as in IL-16 [log(2)-fold change −0.28, *q*-value 0.55], IL1-RAP [log(2)-fold change −0.68, *q*-value 0.28], and TLR2 [log(2)-fold change −0.15, *q*-value 0.89] with values trending toward baseline values (Figure 7). In addition, there were decreases in the same neutrophil-related proteins, such as AZU1 [log(2)-fold change −1.97, *q*-value 0.16], PRTN3 [log(2)-fold change −2.07, *q*-value 0.11], MPO [log(2)-fold change −1.80, *q*-value 0.15], and neutrophil-related elastase [ELANE, log(2)-fold change −1.84, *q*-value 0.15] (Figure 8). There was also an increase, though not statistically significant, in the macrophage-related regulator protein NOSTRIN [log(2)-fold change 0.68, *q*-value 0.24] (Figure 8).

**Table 2:** Proteins identified related to the inflammation and cytokine processes compared between the treated and non-treated group at the end of observation after left lung transplantation.

Protein	Log(2)-fold change	*q*-value
IL-1RAP	−0.68	0.28
IL-16	−0.28	0.55
TLR2	−0.15	0.89
AZU1	−1.97	0.16
PRTN3	−2.08	0.11
MPO	−1.80	0.15
ELANE	−1.84	0.15
NOSTRIN	0.68	0.24

IL-1 RAP, interleukin-1 receptor accessory protein; IL-16, pro-interleukin-16; TLR2, toll-like receptor 2; Azu1, azurocidin; PRTN3, proteinase 3; MPO, myeloperoxidase; ELANE, elastase, neutrophil expressed; NOSTRIN, nitric oxide synthase trafficking.

## Discussion

Given the low utilization of donor organs paired with the high demand, the regeneration and subsequent preservation of otherwise discarded lungs are greatly needed. One methodology for addressing this issue would be treatment with cytokine adsorption, which can be administered during EVLP. However, the biological processes affected by this treatment are not fully characterized. This current study leverages the proteomic analyses performed on lung tissues collected from the lungs treated with cytokine adsorption during EVLP and post-transplantation and non-treated lungs, to better understand this type of graft preservation. The results show that the inflammatory- and immune-related pathways were modulated by the treatment device. The GSEA analysis revealed altered processes related to inflammatory responses, immune responses, stress responses, and leukocyte-mediated immunity pathways. By the end of the observational period after transplantation, the humoral immune response, inflammatory response, and defense response were all significantly downregulated in the treated group compared to those in the non-treated group. This approach of studying treated and non-treated donor lung grafts allowed for a more global understanding of the protein processes affected by cytokine adsorption and showed that the treatment causes broad changes in the inflammatory pathways.

This is important given the existing literature on cytokine adsorption as a treatment that currently focuses on the presence and quantification of particular cytokine levels. In contrast, this study aims to extend our understanding of the treatment effect beyond those parameters to see how larger, more global pathways are affected on a tissue level. As noted, cytokine adsorption has been explored as a treatment modality in the context of EVLP. The adsorber removes the middle- and low-molecular-weight molecules through adsorption to polymer beads, with promising results when applying the adsorber to the treatment of ischemia–reperfusion injury and prolonged EVLP (32–34). For lungs kept in cold ischemia for prolonged periods, such as up to 24 h followed by a longer EVLP duration of 12 h, positive findings of improved compliance and edema have been demonstrated. The integration of the cytokine adsorber in this case allowed for longer periods of cold storage in the lungs that were healthy at the time of acquisition. In our previous study on cytokine adsorption, the adsorber was used to restore lung function in discarded lungs with signs of ARDS to increase the available donor pool. We showed that the treatment improved pulmonary function during EVLP and post-transplantation, along with a decreased incidence of primary graft dysfunction (19). However, in both of these studies that used adsorption for either longer preservation of healthy lungs or preservation of severely damaged grafts, there has not yet been an exploration of the types of molecular processes affected by the treatment. This current study expands on those translational findings to augment them with a more in-depth proteomic evaluation.

Such work is needed given the number of donor lungs that are routinely discarded. Donor grafts may be rejected due to several etiologies, and ALI due to infection is an important cause. To model this type of infection-induced injury, an LPS-induced ARDS injury was utilized in this porcine model. LPS which is an endotoxin derived from *E. coli* results in endothelial damage that ultimately causes lung injury similar to human pathology. In this study, the resulting ARDS that developed after LPS was given would have resulted in lungs typically declined for use in transplant. Instead, these lungs were harvested and then placed on EVLP. The addition of the cytokine adsorber in line with EVLP allowed for an isolated system in which the lungs could be both treated and evaluated. Subsequently, the lungs were transplanted where they then received further treatment with the cytokine adsorber and were found to have improved function when compared with those in the non-treated group.

Among a small number of studies focusing on cytokine adsorption in the context of EVLP, there are none yet to our knowledge that have investigated the proteomic profiles of the treatment. In this study, proteins were identified using mass spectrometry to characterize the proteome found within the tissues. The resulting differential expression of proteins was computed, and the results showed that each group could be clearly distinguished from one another. Furthermore, the overall differences as shown in the heatmaps both after EVLP and lung transplantation follow-up were significant enough to differentiate the treated group from the non-treated group, as well as each from healthy lung tissue.

Transitioning from the holistic view of the proteome, the biological processes were identified by the GSEA analysis to characterize the biological processes found within the identified proteins. This analysis allowed for the observation of activity patterns across the dataset to see the pathways with biological relevance. From this analysis, the pathways related to inflammatory and immune responses were highlighted, as seen in the GSEA dot plots. In EVLP, the GO terms such as “immune system process” and “coagulation” were further examined particularly given the significance of the terms in relation to ARDS. Post-transplantation, an effect of cytokine adsorption, can be appreciated in the treated group, where biological processes such as humoral immune response, external stimulus response, defense response, and stress response were all suppressed. Cytokine adsorption has been tested in studies for its effect on particular individual cytokines, and these results have introduced the hypothesis that the treatment modality has an overall effect on larger immune responses.

In addition, the biological processes related to coagulation and hemostasis were found to be suppressed in the treated group when compared to the non-treated group at the end of observation after transplantation. This suggests a positive effect on coagulation hemostasis, which would be important in both the setting of the ARDS induced in the donor lung in this experiment and in surgical procedures in general, where the risk of bleeding is an important consideration. This model aims to test whether lungs that had experienced sepsis-induced ARDS could be recovered for lung transplantation using cytokine adsorption, which is significant given the number of grafts declined for use due to infection damage. To this point, coagulation dysfunction is a known sequela of ARDS with activation of the coagulation pathway that is known to perpetuate further damage in lung disease (35). Furthermore, states of inflammation further drive pathological clotting (36). The finding of coagulation hemostasis regulation with the treatment is important, particularly since the observed differences in the coagulation-related gene ontology pathways had a lasting effect since the analysis was performed at the end of the 3-day observation period.

To augment the analysis of pathways, individual proteins were observed to look for patterns across proteins involved in inflammation and immune processes. This included decreases in the treated group that were numeric but not statistically significant in proteins linked to inflammation, such as IL16, TLR2, PYCARD, IL-6ST, CMLK1, and cREL. Pro-interleukin-16 served as the precursor to interleukin-16, a pro-inflammatory cytokine that is a chemoattractant and has a direct correlation with the number of infiltrating CD4^+^ T cells (37). Other cytokine-related proteins identified within the study were related to interleukin-1 and interleukin-6. The interleukin-6 cytokine family is defined by their signal transduction through IL-6ST, which was decreased in this study (38). IL-6 is a significant cytokine given its stimulatory effect on B and T cells and its described correlation with worsening morbidity and mortality in human ARDS (38–40). Moreover, IL-1 has been specifically identified as a prognostic indicator of outcomes in lung transplant, with the potential to differentiate donor graft performance (41, 42). RNA levels of IL-6 and IL-1 from human donor lungs were also correlated with increased risk of mortality post-transplantation, supporting the hypothesis that these cytokines and their related pathways are important in mitigating poor outcomes (43). Furthermore, TLR2 is a cell membrane receptor that, similar to other toll-like receptors, recognizes pathogen molecular patterns or pathogen associated molecular pattern (PAMPs) and activates immune cells after PAMP detection. TLR2 has a wide range of PAMP detection including Gram-positive and Gram-negative bacteria and was observed in lower amounts in the treated group in this study (44). The decreases from within the treated group relative to the non-treated group were observed during both EVLP and the post-transplantation period. This points to a sustained effect, particularly given that the cytokine adsorption treatment was given immediately post-transplantation while the lung biopsies were acquired after 3 days of observation.

In terms of proteins related specifically to neutrophils and macrophages, there were several related identities singled out for comparison. Azurocidin, proteinase 3, neutrophil elastase, and myeloperoxidase were detected across the three groups, with modestly lower values in the treated group. These proteins are involved in neutrophil degranulation and neutrophil extracellular traps (45–47). DPP1 plays a role in neutrophil maturation as it activates serine proteinases, and inhibitors of DPP1 have been explored in lung disease as a method of decreasing neutrophil activity (48). The reduction of neutrophilic involvement is a key target in a lung transplantation setting, given the known contribution of neutrophil extracellular traps (NETs) to pathological states with worse post-transplant outcomes (20, 49). In addition, platelet basic protein is a neutrophil chemoattractant and activator, with increasing levels shown to correlate with other forms of lung disease (50).

In proteins related to macrophage function and identity, the macrophage marker CD163 was detected, and a decrease was appreciated between the treated and non-treated groups. Moreover, nitric oxide synthase catalyzes the production of nitric oxide needed by macrophages for their oxidative burst. On the other hand, the NOSTRIN is a regulator of nitric oxide, resulting in attenuation of its production through sequestration in endothelial cells and inhibited adhesion of macrophages (51). In this study, NOSTRIN was observed at higher values in the treated group compared to the baseline and non-treated groups, both at the end of EVLP and post-transplantation, which implies the presence of different angles from which macrophage involvement is regulated with the treatment. Collectively, the pattern observed both after EVLP and post-transplantation demonstrates that neutrophils and macrophages are affected by the cytokine adsorption treatment. Neutrophils are known to be mediators of inflammation, with NETs contributing to the escalation of inflammatory responses, thrombogenesis, and damage to lung tissue, which manifests as primary graft dysfunction in lung transplantation (49). In addition, the regulation of macrophages is an important process to control within lung transplantation, given the known associations with escalating inflammation and donor macrophages to worsening reperfusion injury (52–54). The finding of reduced markers and effector proteins from within these two immune cell types in the treated group points to an effect of the cytokine adsorber that implies a broader effect. The reduction of cytokines has been explored in other studies when using the cytokine adsorber; however, this study demonstrates that the consequences of the treatment can be more expansive on the immune system and its associated pathways.

The extended effect of the cytokine adsorption treatment can be appreciated when looking at the proteomic changes from the individual level up through the biological processes and then beyond when seeing the changes in overall protein expression profiles within the identified proteome. We observed first that there were global changes that clearly distinguished the treated group from the non-treated group and that further distinguished the treated group from the group with healthy lungs, both after treatment during EVLP and post-transplantation. Furthermore, immune, inflammatory, and defense processes were observed to change throughout the experimental timeline, and the treatment had a further effect on the impact of coagulation, particularly seen post-transplantation. This demonstrates the important effects of the therapy on processes that extend beyond the examination of individual cytokine levels. As cytokine adsorption emerges as a promising therapy for the recovery of marginal and discarded lungs in transplantation, an understanding of the processes that underlie its efficacy is important. We demonstrate here the overarching effects that show that the treatment modulates the immune, inflammatory, and coagulation pathways to change the response in discarded lungs. The findings of this study augment the clinical and histopathological improvements previously seen within studies on cytokine adsorption in line with EVLP and demonstrate the efficacy of using the treatment in graft preservation.

## Data Availability

The datasets presented in this study can be found in online repositories. The names of the repository/repositories and accession number(s) can be found in the article.
